# X-ray ghost imaging with a specially developed beam splitter

**DOI:** 10.1107/S1600577524008038

**Published:** 2024-09-30

**Authors:** Chang-Zhe Zhao, Hai-Peng Zhang, Jie Tang, Ni-Xi Zhao, Zhong-Liang Li, Ti-Qiao Xiao

**Affiliations:** a Shanghai Institute of Applied Physics Chinese Academy of Sciences Shanghai201800 People’s Republic of China; b Shanghai Synchrotron Radiation Facility, Shanghai Advanced Research Institute Chinese Academy of Sciences Shanghai201204 People’s Republic of China; c University of Chinese Academy of Sciences Beijing100049 People’s Republic of China; Australian Synchrotron, Australia

**Keywords:** X-ray ghost imaging, X-ray beam splitter, synthetic aperture X-ray ghost imaging, speckle correlation of X-rays

## Abstract

X-ray ghost imaging with a crystal beam splitter has advantages in highly efficient imaging due to the simultaneous acquisition of signals from both the object beam and reference beam. However, beam splitting with a large field of view, uniform distribution and high correlation has been a great challenge up to now. Therefore, a dedicated beam splitter has been developed by optimizing the optical layout of a synchrotron radiation beamline and the fabrication process of a Laue crystal. A large field of view, consistent size, uniform intensity distribution and high correlation were obtained simultaneously for the two split beams. Modulated by a piece of copper foam upstream of the splitter, a correlation of 92% between the speckle fields of the object and reference beam and a Glauber function of 1.25 were achieved. Taking advantage of synthetic aperture X-ray ghost imaging (SAXGI), a circuit board of size 880 × 330 pixels was successfully imaged with high fidelity. In addition, even though 16 measurements corresponding to a sampling rate of 1% in SAXGI were used for image reconstruction, the skeleton structure of the circuit board can still be determined. In conclusion, the specially developed beam splitter is applicable for the efficient implementation of X-ray ghost imaging.

## Abstract 2

Compared with traditional imaging methods, the non-local characteristics of ghost imaging have the ability to greatly reduce the radiation dose of X-ray imaging in principle, and has important application prospects in radiation-sensitive fields such as biomedicine. Here a specially developed beam splitter applicable for the efficient implementation of X-ray ghost imaging is described.

## 1. Introduction

Owing to its nonlocal imaging characteristics, ghost imaging (GI) has been widely used for imaging with visible light, infrared, X-ray and particle waves (Cheng & Han, 2004 ▸; Liu & Zhang, 2017 ▸; Li *et al.*, 2018 ▸; He *et al.*, 2021 ▸). In addition, it can be implemented with few photons (Lane & Ratner, 2020 ▸). X-rays have unique advantages due to their short wavelength and strong penetration, but they also cause ionization damage to samples. Compared with traditional imaging methods, the nonlocal characteristics of GI have the potential to greatly reduce the radiation dose of X-ray imaging in theory and have important application prospects in radiation-sensitive fields such as biomedicine (Ceddia & Paganin, 2018 ▸). The experimental validation of X-ray ghost imaging (XGI) has made important progress in recent years (Olbinado *et al.*, 2021 ▸; Klein *et al.*, 2022 ▸; Ceddia *et al.*, 2023 ▸), but there are still problems such as a low signal-to-noise ratio, low data acquisition efficiency, difficulty achieving high resolution, a large field of view and a low radiation dose, which seriously restrict the practical applications of this method.

GI realizes image reconstruction based on the intensity correlation of two beams with consistent fluctuation characteristics. Obtaining the two beams in the object arm and reference arm with high correlation is the key to GI experiments. Unlike with visible light, beam splitting of X-rays is much more difficult. The virtual beam splitting scheme can avoid the actual splitting of X-rays, which enables signal acquisition of the object beam and the reference beam by switching over the sample in the incident X-ray beam or obtaining the reference signals directly via computational GI (Yu *et al.*, 2016 ▸; Zhang *et al.*, 2018 ▸; He *et al.*, 2020 ▸). However, this scheme needs to switch the sample into and out of the X-ray beam to achieve data acquisition of the reference and object signals, respectively, which reduces the efficiency of the method greatly. Therefore, high-quality beam splitting is critical for the efficient implementation of XGI, in which a large and consistent beam size, uniform intensity distribution and high correlation of the two split beams are required.

XGI with a crystal-based beam splitter has been reported, including experimental studies based on synchrotron radiation sources (Pelliccia *et al.*, 2016 ▸, 2018 ▸; Kingston *et al.*, 2018 ▸) and laboratory X-ray sources (Schori & Shwartz, 2017 ▸). Kunimune *et al.* used monocrystalline silicon Laue diffraction to split a monochromatic synchrotron X-ray beam and first observed X-ray correlation of monochromatic synchrotron X-rays (Kunimune *et al.*, 1997 ▸). Pelliccia *et al.* (2018 ▸) and Kingston *et al.* (2018 ▸) used natural masks to generate speckle patterns and then performed beam splitting using double-sided polished monocrystalline silicon wafers. However, problems such as low correlation between the diffracted beam and transmitted beam and inconsistent spot size prevented it from achieving XGI higher image quality. Zhao *et al.* (2022 ▸, 2024*a* ▸) successfully improved the spatial correlation of two beams based on the dynamic theory of X-ray diffraction. The lattice of the crystal for X-ray diffraction is in the angstrom region, which means that any slight variation in the incident beam and the crystal itself will deteriorate the efficiency of the beam splitting. Therefore, developing a dedicated crystal beam splitter for XGI is highly important.

To meet the demand of XGI with a beam splitter, this paper reports the development of a dedicated crystal beam splitter at the Shanghai Synchrotron Radiation Facility (SSRF) and experimentally verifies the feasibility of its application for XGI. First, the optimization of the beam splitter is introduced. Then, an experiment with a circuit board is carried out to demonstrate the applicability of the beam splitter for XGI. With the aim of obtaining low-dose X-ray imaging, XGI with fewer measurements has also been investigated. Finally, conclusions and related discussion are provided.

## 2. Beam splitter optimization for XGI

By optimizing the optical setup of the X-ray beamline to maintain the lattice plane of the Bragg crystal of the monochromator and Laue crystal of the beam splitter in a nondispersive configuration (see Appendix *A*[App appa]), the receiving angle of the crystal of the beam splitter needs to match the divergence angle of the incident X-ray beam from the double-crystal monochromator to ensure the consistency of the field of view between the object (diffraction) beam and the reference (transmission) beam. Both the monochromator crystals and the Laue crystal adopt low indices of the lattice plane for X-ray diffraction to ensure the high-flux output of the beam, increase the number of photons for XGI, and improve the imaging signal-to-noise ratio and data acquisition efficiency. The Laue crystal is manufactured as a whole with high-quality crystal rods grown by floating zone technique to avoid processing stress and clamping stress affecting the uniformity of the split beams.

**Figure 1:**
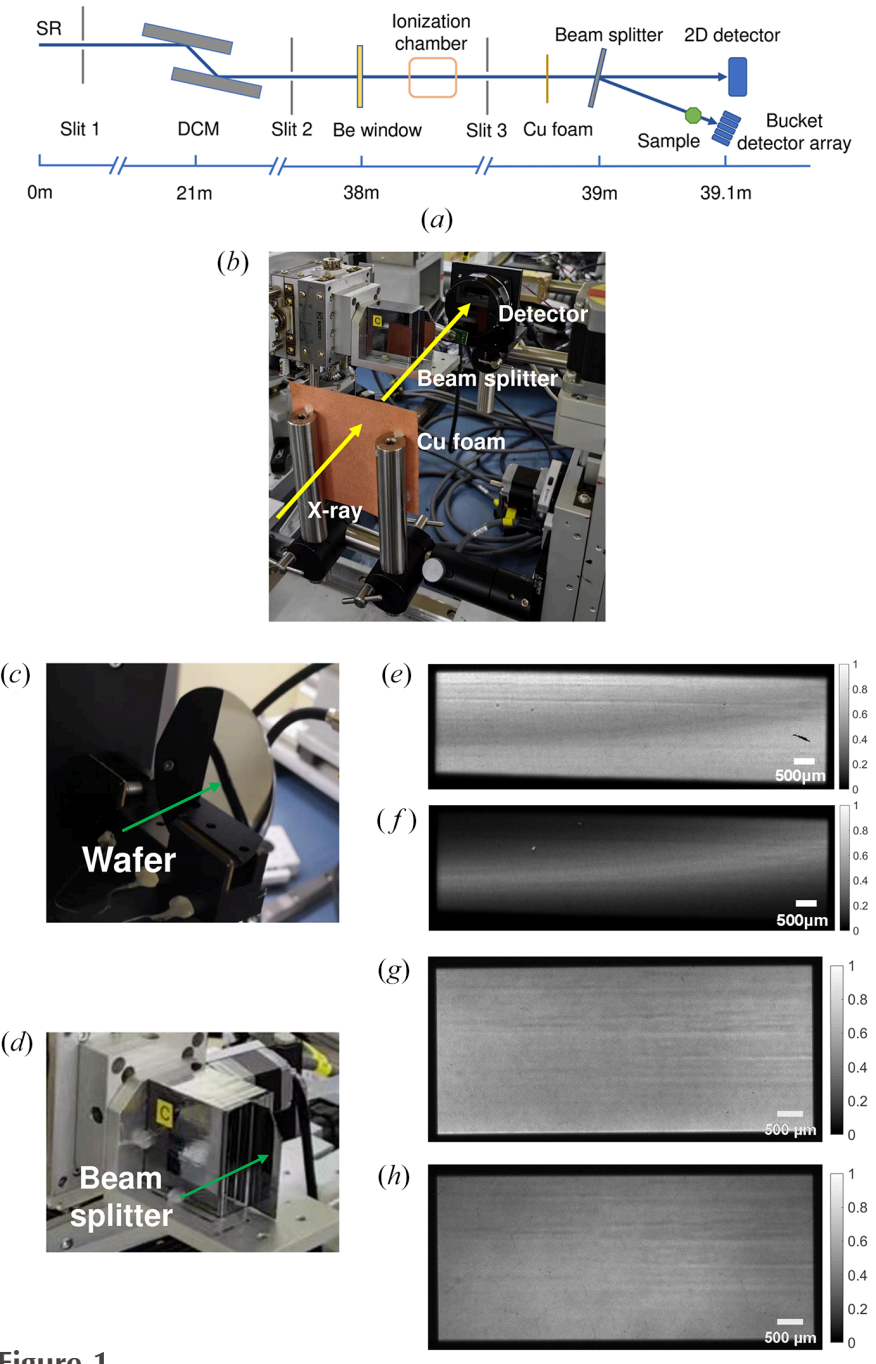
(*a*) Schematic diagram of the X-ray beamline, (*b*) experimental setup for XGI, (*c*) photograph of the wafer, (*d*) photograph of the specially developed beam splitter, (*e* and *f*) transmission and diffraction beams of a conventional wafer, and (*g* and *h*) transmission and diffraction beams of the dedicated beam splitter.

Experiments were carried out at the test beamline BL09B of the SSRF. The beamline utilizes hard X-rays from a bending magnet for at-wavelength metrology and crystal characterization (Li *et al.*, 2019 ▸). Fig. 1 ▸(*a*) shows a schematic diagram of the optical setup of the beamline. A Si(111) double-crystal monochromator (DCM) is utilized to monochromatize the white light from the bending magnet of the storage ring. Considering the penetration through the copper foam for intensity modulation, a photon energy of 20 keV was selected for the experiments. The thickness of the copper foam is approximately 300 µm, with pore sizes ranging from 150 to 190 µm and an average porosity of 77.4%. According to the results obtained using a multi-crystal configuration (Yang *et al.*, 2020 ▸), the divergence angle of a monochromatic X-ray beam is approximately 26 arcsec, and the relative energy bandwidth is 6.3 × 10^−4^. Considering the strong absorbing characteristics of copper foam, the beam intensity is randomly modulated to generate speckle patterns for XGI. A specially manufactured Laue crystal was employed to split the incident monochromatic X-ray beam for the DCM into diffraction and transmission beams. Then, the two split beams with an angle of 5.67°, *i.e.* the reference and object beams of XGI, were recorded by a pixel array detector (OnSemi KAI-16000, an X-ray CCD camera) simultaneously, immediately downstream of the sample. Considering the small angle between the split beams, the use of one pixel-array detector to record the two beams at the same time will not cause significant errors. One part of the detector acts as a two-dimensional detector for the reference beam, while the other part is taken as a bucket detector array of synthetic aperture X-ray ghost imaging (SAXGI) (Zhang *et al.*, 2022*a* ▸). The effective area of the detector is 36 mm × 24 mm with a pixel size of 7.4 µm. As shown in Fig. 1 ▸(*a*), a set of slits is used to define the beam size and reduce the effect of X-ray scattering on the imaging signal-to-noise ratio. Slit 1 installed in the vacuum chamber is mainly used to define the incident beam size. Slit 2 inside the vacuum and Slit 3 outside the vacuum are used to block the scattered X-rays. Behind the beryllium window used for isolating the beamline vacuum from the atmosphere, a gas ionization chamber is used to monitor the real-time intensity of the incident monochromatic X-ray beam. Due to the limited distance between the beryllium window and the detector, the absorption loss of the 20 keV X-rays in the atmosphere is almost negligible.

As shown in Fig. 1 ▸(*b*), a Laue crystal of the dedicated beam splitter, located 10 cm downstream of the copper foam, was used to split the incident beam into two beams by Laue diffraction. To ensure the consistency of the imaging field of view in the split beams, we made efforts in two aspects, including the nondivergence match of crystal optics between the DCM and the splitter, and strain elimination of the Laue crystal. The monochromatic beam mentioned above has a divergence angle of 26 arcsec, which is much greater than the acceptance angle of the Si(111) crystal (2.7 arcsec). This disparity is a result of the coupling between the photon energy and divergence angle, where the angular distribution reflects the photon energy distribution. The distribution of photon energy can result in inconsistencies in the imaging fields of view between the transmitted beam and diffracted beam. To eliminate the detrimental effects caused by the energy distribution, a Laue diffraction crystal is used as a beam splitter with the same lattice plane as the DCM Si(111), for which the Bragg angle is 5.67°. Simultaneously, the lattice planes of the DCM and beam splitter are aligned parallel to each other in a dispersion configuration. Thus, the acceptance angle of the beam splitter is matched with the divergence angle of the monochromatic beam from the DCM, which ensures consistent imaging fields of view for both the transmitted and diffracted beams. For both the DCM and beam splitter, a low-index lattice plane is selected for crystal diffraction to ensure a high flux output for XGI and a high correlation between the two beams (Zhao *et al.*, 2024*a* ▸).

The strain of the Laue crystal will lead to an uneven intensity distribution in the split X-ray beam. At the initial stage, we used a piece of wafer with a thickness of 300 µm and both faces polished to split the X-ray beam, as shown in Fig. 1 ▸(*c*). The fixation method of clamping inevitably leads to stress inside the crystal, and the corresponding strain of the lattice results in a distorted beam output. Figs. 1 ▸(*e*) and 1(*f*) show transmission and diffraction beams of the wafer processed via a conventional procedure and fastened by clamping. Obviously, the uniformity of the two beams is severely deteriorated due to the stress resulting from crystal clamping. The distortion of the crystal lattice plane at the scale of the lattice constant leads to an apparent uneven intensity distribution. It is difficult to improve the output beam by optimizing the clamping style.

To address this problem, a dedicated beam splitter was fabricated from a floating-zone silicon single-crystal ingot to avoid the processing and clamping stress of the beam splitter (Zhao *et al.*, 2024*b* ▸). As shown in Fig. 1 ▸(*d*), the developed beam splitter consists of a working area and a base. The effective working area is 15 mm × 40 mm with a thickness of 300 µm after acid etching, while the base is used for supporting without clamping during the experiments. By compromising the correlation and structural rigidity of the crystal plate, a thickness of 300 µm is selected (Zhao *et al.*, 2024*a* ▸). As shown in Figs. 1 ▸(*g*) and 1(*h*), transmission and diffraction beams of the same size are obtained by the specially developed beam splitter. Slight irregularities in the intensity distribution can also be observed, which reflect the uneven thickness of the working area limited by the fabrication precision. The developed beam splitter ensures that every point at the surface of the Laue crystal satisfies the same diffraction conditions for an incident X-ray beam, thus achieving a consistent beam size after splitting.

**Figure 2:**
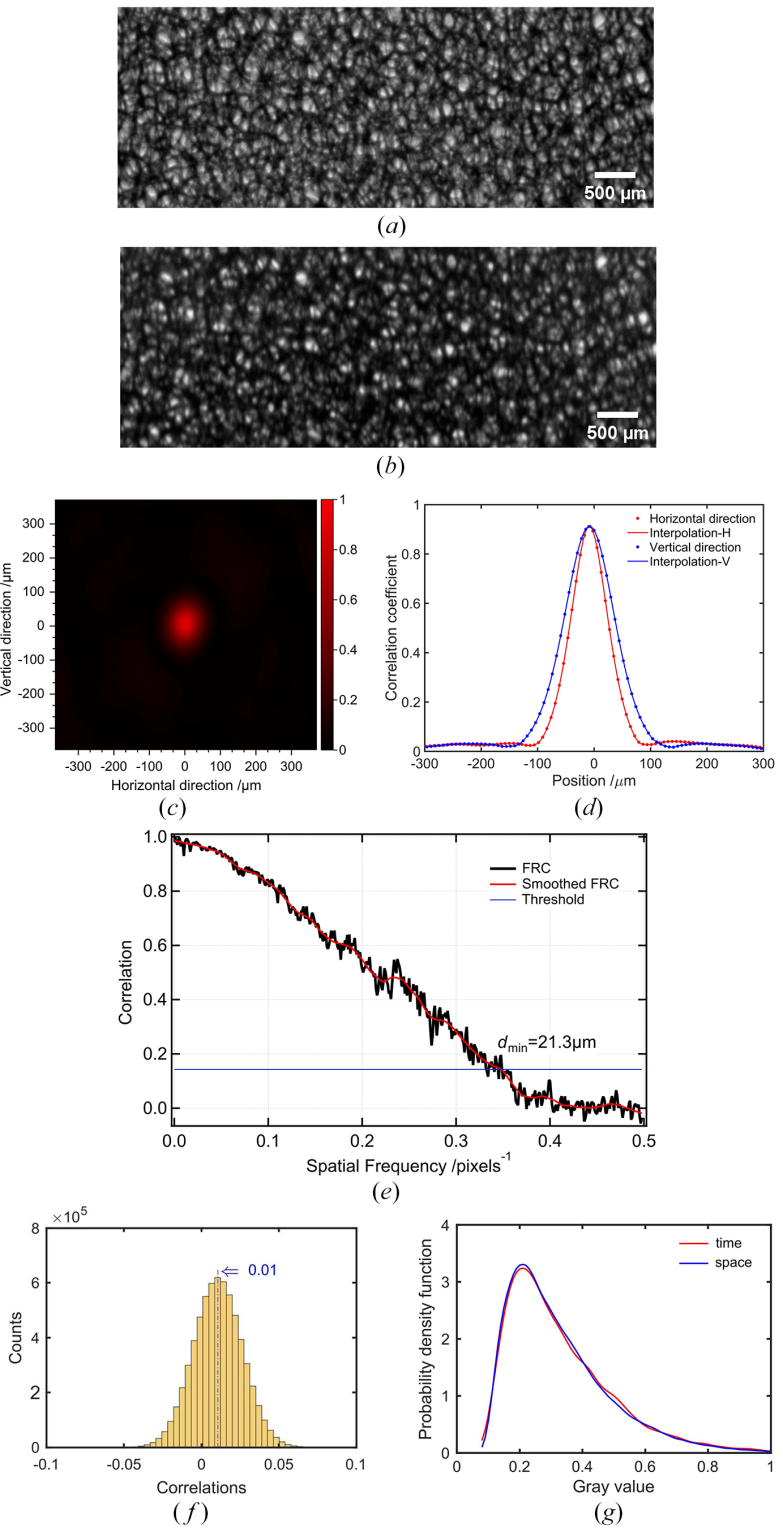
Correlation characteristics of the split speckle patterns. (*a*) Reference beam; (*b*) object beam; (*c*) point spread function (PSF) of GI; (*d*) central line-profile of the PSF in the horizontal and vertical directions; (*e*) FRC between the reference and object speckle patterns; (*f*) correlation statistical histogram between different speckle patterns; and (*g*) statistical distribution in the spatial and temporal dimensions.

To evaluate the applicability of the developed beam splitter for XGI, the correlation characteristics between two split beams randomly modulated by a piece of copper foam were investigated. The results are shown in Fig. 2 ▸. Fig. 2 ▸(*a*) shows one of the speckle patterns in the reference beam, while Fig. 2 ▸(*b*) shows one of the speckle patterns in the corresponding object beam. The intensity of the transmitted beam is approximately three times stronger than that of the diffracted beam. The imaging fields of view after beam splitting are uniform and consistent with the preserved details. We achieved beam splitting with spatial intensity correlation, which is a prerequisite for XGI. The reconstruction of the GI can be obtained with the following formula (Bromberg *et al.*, 2009 ▸; Katz *et al.*, 2009 ▸),
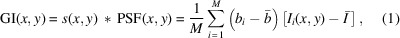
where *x*, *y* are the coordinates on the detection plane for the sample image *s*(*x*, *y*), which contains *n* pixels; * denotes convolution over *x* and *y*; PSF is the point-spread function; *M* is the total number of measurements; *b*_*i*_ is the total intensity value of the *i*th measurement in the object arm; *I*_*i*_(*x*, *y*) is the speckle pattern of the *i*th measurement in the reference arm; and 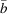 and 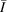 are the average intensities of all the *M* measurements in the object arm and reference arm, respectively. PSF is associated with finite spatial resolution. The covariance of the speckle fields defines the PSF of GI, which implies a spatial resolution comparable with the smallest characteristic size in speckle fields. Accordingly, the correlation coefficient is the normalized form of the covariance. Therefore, the resolution of the GI can be approximately estimated by the two-dimensional correlation coefficient,
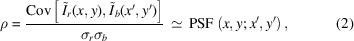

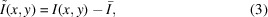
where ρ is the two-dimensional correlation coefficient, subscripts *r* and *b* represent the reference arm and object arm, respectively, Cov represents covariance, and σ is the standard deviation.

The peak of the two-dimensional correlation coefficient represents the normalized correlation of the split beams, and the full width at half-maximum (FWHM) of the peak can estimate the spatial resolution of GI (Pelliccia *et al.*, 2018 ▸; Oh *et al.*, 2013 ▸; Sprigg *et al.*, 2016 ▸). According to equation (2), the calculated values of the PSF are shown in Fig. 2 ▸(*c*), and Fig. 2 ▸(*d*) shows the central line-profile of the PSF in Fig. 2 ▸(*c*). The calculations were performed by randomly selecting five pairs of beam-splitting images and taking the average. As a result, the correlation coefficient between the two modulated beams is significantly improved to 0.92, while the value for the split beams from a wafer is 0.52 (see Appendix *B*[App appb]). The deterioration of the correlation is related to the characteristics of the incident beam, beam splitting crystal and modulator, and more details are given in §4. According to Fig. 2 ▸(*c*), the spatial resolution for XGI is slightly anisotropic in two dimensions, with lower resolution in the diffraction direction due to the effect of dynamic diffraction. According to the profile shown in Fig. 2 ▸(*d*), the FWHM is about 76 µm in the horizontal direction and about 104 µm in the diffraction direction (vertical direction) on average. We also used Fourier ring correlation (FRC), applied to registered speckle patterns, to estimate a best-case limit for the spatial resolution of GI (Kingston *et al.*, 2018 ▸). As shown in Fig. 2 ▸(*e*), at a fixed 1/7 FRC, the corresponding value of 21.3 µm is the upper limit of the spatial resolution that can be achieved by GI. At present, the resolution has not reached the upper limit, which may be related to the larger size of the beam modulator.

We utilized copper foam, a strong-absorbing material, to generate speckle patterns with high contrast for the random modulation of XGI. An ensemble of speckle patterns for XGI was obtained by a raster scan of the copper foam. The step size of the lateral displacement of 200 µm was larger than the FWHM of the PSF to ensure randomness between speckle patterns. To quantify the randomness of the ensemble of speckle patterns, correlation coefficients were calculated for 3600 speckle patterns randomly selected from 12000 speckle patterns. A total of 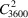 calculations were performed, and the statistical results are shown in Fig. 2 ▸(*f*). The correlation coefficient between all combinations of the speckle pattern ensemble is 0.01 ± 0.02, which indicates a low correlation between the randomly modulated speckle patterns. In addition, the statistical properties of this random process were analyzed. The gray values of all the pixels in a single speckle pattern and the gray values of a pixel in all 12000 speckle patterns were selected for statistical analysis via a probability density function. As an artificial pseudothermal light field, the gray value fluctuation of a certain pixel in all the collected speckle patterns imitates the evolution of the thermal light field with time. As shown in Fig. 2 ▸(*g*), the spatial statistical distribution in a speckle pattern is in high agreement with the temporal distribution, which indicates that the speckle fields generated by the copper foam are ergodic. To further evaluate the intensity fluctuation of the speckle field, the normalized second-order correlation function is utilized,
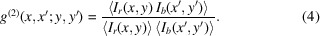
The calculated value of *g*^(2)^(*x*, *x*′; *y*, *y*′) is 1.25, which indicates that the speckle field exhibits high fluctuation properties and a strong noise resistance capability. As a result, the quantitative characterization of the speckle field generated by a natural mask of copper foam in terms of randomness, ergodicity and fluctuation demonstrates that the artificial pseudothermal light field generated is compliant with the requirements of XGI.

## 3. X-ray ghost imaging with split beams

SAXGI (Zhang *et al.*, 2022*a* ▸) is introduced to make full use of the advantages of the large size of the split beam with a lower sampling rate, in which the bucket detector in conventional GI was replaced by a bucket detector array. The objective of this experiment is to evaluate the quality of split X-ray beams for ghost imaging. To avoid the effect of alignment errors on the image reconstruction through the correlation between the split beams, the object beam and reference beam are recorded without pixel binning by different areas of a single X-ray CCD detector. During image reconstruction, signals in the object beam are binned accordingly to form a so-called bucket detector array to meet the requirements of SAXGI. Usually, the binned area is much smaller than the beam size itself and fewer measurements are required to maintain a certain sampling rate.

**Figure 3:**
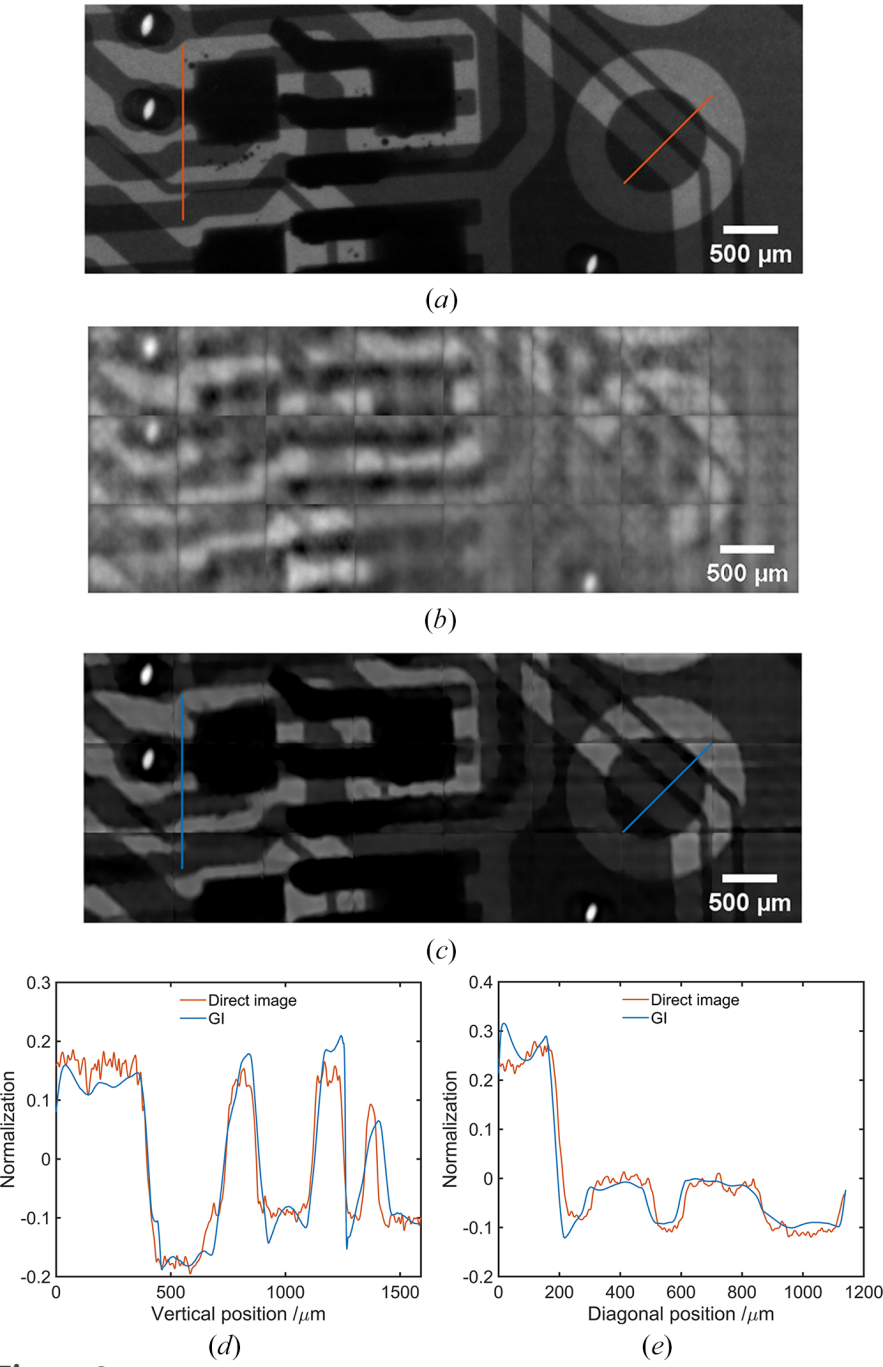
X-ray ghost imaging of a circuit board. (*a*) Projection image with a size of 880 × 330 pixels; (*b*) GI reconstruction using the ensemble averaging algorithm, where the bucket size for SAXGI is 110 × 110 pixels and the number of measurements is *M* = 11234; (*c*) GI reconstruction using the compressed sensing algorithm, with the same bucket size and measurement number; normalized line profiles for weak absorption (*d*) and strong absorption (*e*) circuit components at positions denoted by red and blue lines in (*a*) and (*c*), respectively.

A circuit board is employed for the XGI experiments, in which weak and strong absorption circuit materials are used to evaluate the ability of XGI to reveal structures with different X-ray contrasts. We conducted validation experiments at BL09B of the SSRF, and Fig. 3 ▸ shows a comparison between ghost imaging and traditional projection imaging. In the experiment, we used the same pixel array detector (7.4 µm pixel^−1^) to simultaneously collect the signals from the object and reference beam. The exposure time was set to 50 ms based on the grayscale achieved in the reference beam. Fig. 3 ▸(*a*) shows a direct projection image of the circuit board with an image size of 880 × 330 pixels. Fig. 3 ▸(*b*) shows the result of GI reconstruction by ensemble averaging, which has a low signal-to-noise ratio and a significant block effect due to pixel binning in the object beam. Therefore, image reconstruction with SAXGI is employed to improve image quality. According to the principle of SAXGI, the signals in the object beam were effectively converted into a bucket detector array by pixel binning. The image reconstruction algorithm of compressed sensing was based on TVAL3 (Li *et al.*, 2013 ▸), which has strong noise resistance. Fig. 3 ▸(*c*) shows the reconstruction result of compressed sensing GI with a measurement number of *M* = 11234. The bucket size is 110 × 110 pixels, corresponding to a sampling rate of 92.8%. Although there is some deterioration in the structural details, the image reconstructed by SAXGI with compressed sensing is comparable with the direct projection image overall, and the circuit structure is clearly revealed. Furthermore, as shown in Figs. 3 ▸(*d*) and 3 ▸(*e*), the comparison between the GI image and projection image confirms the results for Fig. 3 ▸(*c*). Overall, the profiles of the weak and strong absorption circuit structures revealed by these two imaging methods are consistent. The experiments also demonstrate the significant advantage of compressed sensing compared with the ensemble averaging algorithm when combined with SAXGI. Accordingly, the imaging results verify that the developed beam splitter is applicable for efficient data acquisition and high-quality image reconstruction of XGI.

## 4. Discussion

**Figure 4:**
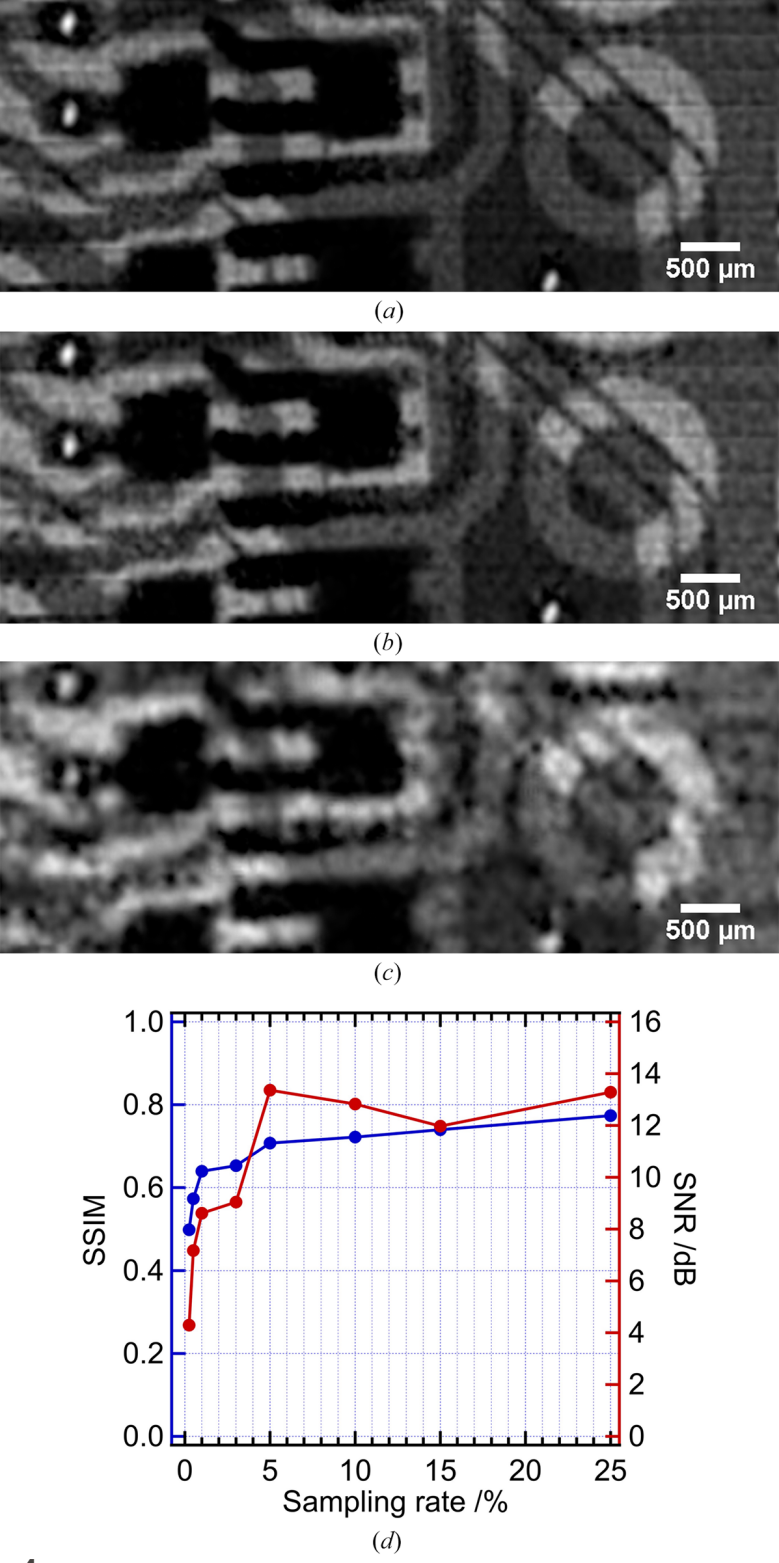
The reconstructed XGI image of 880 × 330 pixels with low measurement numbers where the bucket size is 40 × 40 pixels. (*a*) Measurement number *M* = 160 and sampling rate 10%; (*b*) measurement number *M* = 80 and sampling rate 5%; (*c*) measurement number *M* = 16 and sampling rate 1%; (*d*) SSIM and SNR as a function of sampling rate.

We reduced the number of measurements to investigate the potential of XGI for low-dose X-ray imaging with the developed beam splitter. In SAXGI, the smaller the bucket size, the fewer measurements required for efficient image reconstruction (Zhang, 2023 ▸). By compromising the image quality and number of measurements, a single bucket size of 40 × 40 pixels is selected for the image reconstruction of XGI, and the results are shown in Fig. 4 ▸, in which 1600 measurements give a sampling rate of 100%. Fig. 4 ▸(*a*) shows the reconstructed image of XGI at a sampling rate of 10% (corresponding to 160 measurements) using the compressive sensing algorithm. The overall structure of the circuit board appears relatively intact, with a structural similarity index measure (SSIM) (Wang *et al.*, 2004 ▸) value of 0.72 compared with that of the conventional projection image and a signal-to-noise ratio (SNR) value of 13.29 dB. We then further reduced the number of measurements to 80, corresponding to a sampling rate of 5%. The reconstructed image is shown in Fig. 4 ▸(*b*), in which the overall circuit structure is still distinguishable with an SSIM value of 0.70 but the image contrast is relatively low due to the insufficient sampling rate. Fig. 4 ▸(*c*) displays the result of XGI image reconstruction using only 16 measurements. Although the details are almost overwhelmed by noise, the skeleton of the circuit structure can still be constructed with a SSIM of 0.64 and a SNR of 8.61. As shown in Fig. 4 ▸(*d*), the SSIM and SNR curves jump at a sampling rate of 1%. Thus, the image quality with a sampling rate of 1% essentially reaches the limit of this imaging system. The sampling rate of XGI is directly related to the radiation dose based on the assumption of a consistent pattern illumination fraction per measurement (He *et al.*, 2020 ▸; Ceddia & Paganin, 2018 ▸). Considering that the photon flux of the object beam is one-third of that of the reference beam after the beam splitter, the radiation dose received by the sample is anticipated to be 0.33% of that in conventional projection imaging. Certainly, a low radiation dose can be realized only if two detectors with different sensitivity are employed to record the reference and object signals, respectively. Moreover, the radiation dose of the sample can be further reduced by using a detector with higher efficiency in the object beam.

Although reconstruction of sample information has been achieved with low measurement numbers, there is still much room for improvement in image quality. To obtain a deeper insight into our dual-beam experimental scheme, including its limitations and future opportunities for application, we discuss the characteristics of the beam splitter and the mask. First, we address the impact of the beam splitter on GI. The PSF of dual-beam XGI can be rewritten from equation (2) as follows,
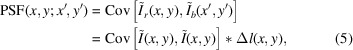

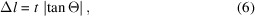
where 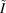 = 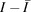; 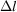 is the position shift of the split X-ray beams; *t* is the thickness of the crystal; and Θ is the angular deviation of the energy flux direction within the crystal, which is closely related to the divergence angle of the incidence beam. This leads to a decrease in the spatial resolution of GI (Zhao *et al.*, 2024*a* ▸). Due to the effect of the incident beam divergence, crystal quality and mask properties, a correlation coefficient of 0.92 for the two split X-ray beams is currently achieved. Using asymmetric diffraction crystals as a beam collimator to obtain highly collimated monochromatic beams (Kuriyama & Boettinger, 1976 ▸), it is feasible to effectively reduce the displacement Δ*l* of the output beam position, thereby improving the spatial resolution of GI. According to the optical setup used in this experiment, when the divergence angle of the incident beam is less than 0.7 arcsec, Δ*l* is smaller than the one-pixel size (7.4 µm), and the diffraction effect has no impact on the speckle pattern. As a result, the effect of crystal diffraction on the spatial resolution of XGI is eliminated.

It is also crucial to select masks that are compatible with the beam splitter. In this work, a natural mask (copper foam) was used to effectively reduce the scattering effects on crystal diffraction, and it exhibited desirable fluctuations of *g*^(2)^ = 1.25 and a randomness of 0.01. However, as shown in Fig. 2 ▸(*f*), the randomness of the speckle patterns follows a Gaussian distribution with a mean of 0.01. Deviations from randomness of zero introduce noise and are unfavorable for image reconstruction of XGI. Aminzadeh *et al.* designed a series of random binary and orthogonal patterns, fabricated with a combination of photolithography and gold electroplating techniques (Aminzadeh *et al.*, 2023 ▸). Such masks, developed to generate high-quality speckle patterns, will not only contribute to image reconstruction but also suppress scattered light, which is beneficial for image reconstruction with beam correlations.

Certainly, high-quality correlated speckle patterns are only one aspect, and the development of image reconstruction algorithms is equally important. In addition to compressed sensing (Kang *et al.*, 2015 ▸; Zhang *et al.*, 2022*b* ▸) and conventional regularization algorithms (Pelliccia *et al.*, 2016 ▸; Kingston *et al.*, 2018 ▸; Zhang *et al.*, 2014 ▸, 2022*c* ▸), deep learning has also played a significant role in image reconstruction (Shimobaba *et al.*, 2018 ▸; Zhu *et al.*, 2020 ▸). Additionally, a global reconstruction strategy can be employed to reduce the impact of block artifacts in SAXGI, but the perfect reconstruction of sub-images remains the ultimate solution for addressing block effects. To achieve the goal of low-dose GI, joint efforts are needed, relating to the experimental setup, speckle properties and image reconstruction algorithms. However, the experimental results demonstrated the potential of the setup and method developed in this paper for the efficient implementation of X-ray ghost imaging, which is an important step toward low-dose X-ray imaging.

## 5. Conclusion

To meet the demand for X-ray ghost imaging with a beam splitter, we developed a dedicated crystal beam splitter at the SSRF and experimentally verified the feasibility of its application for X-ray ghost imaging. By optimizing the optical setup of the X-ray beamline and the beam splitter in a dispersive layout, a consistent field of view of the object beam and the reference beam was achieved. Low indices of the lattice plane for X-ray diffraction were adopted to ensure the high-flux output of the beam splitter and correspondingly improve the correlation between the reference beam and the object beam. The Laue crystal was manufactured using an optimized process to avoid clamping stress, and then intensity uniformity of the split beams was achieved. Combined with a natural modulator of copper foam, the developed beam splitter generated two separate beams with sufficiently large values of the Glauber function for the reconstruction of XGI. The concept of SAXGI is introduced to make full use of the large size of split beams and reduce the sampling of XGI. Finally, experiments on a circuit board demonstrated that the specially developed beam splitter complies with the efficient implementation of XGI. Although there are many aspects to be improved, the method established in the paper lays an important foundation for further extended application of XGI.

## Figures and Tables

**Figure 5:**
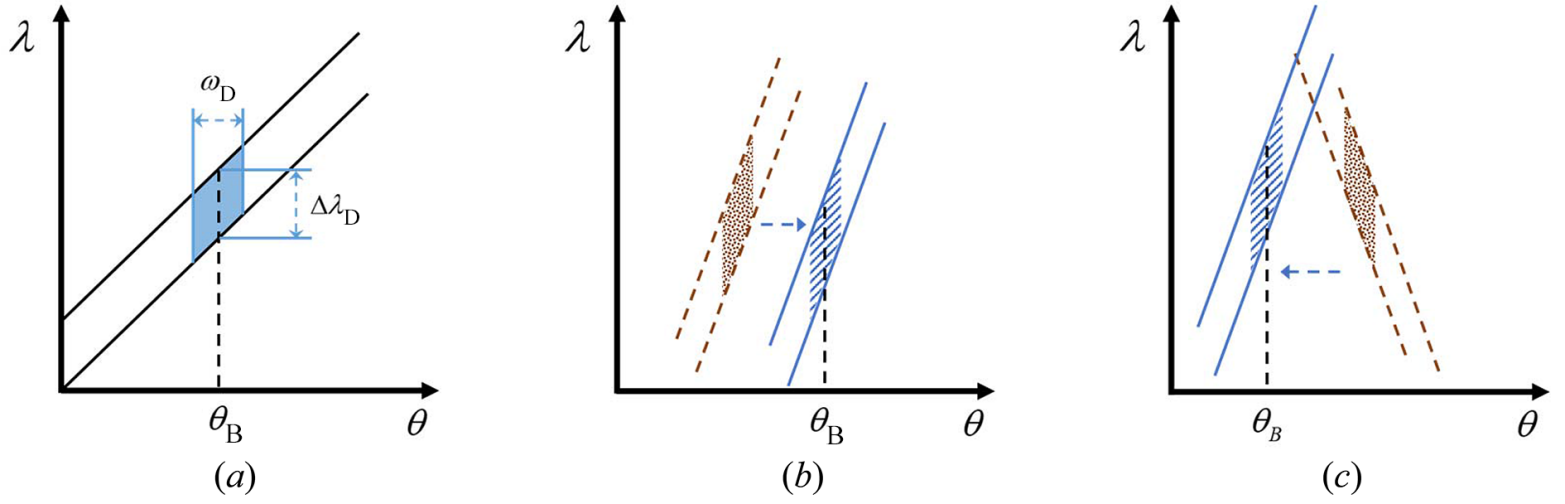
DuMond diagrams for (*a*) perfect crystal diffraction; (*b*) a nondispersive configuration; (*c*) a dispersive configuration. The oblique line and point regions are the DuMond windows of the monochromator and beam splitter, respectively.

**Figure 6:**
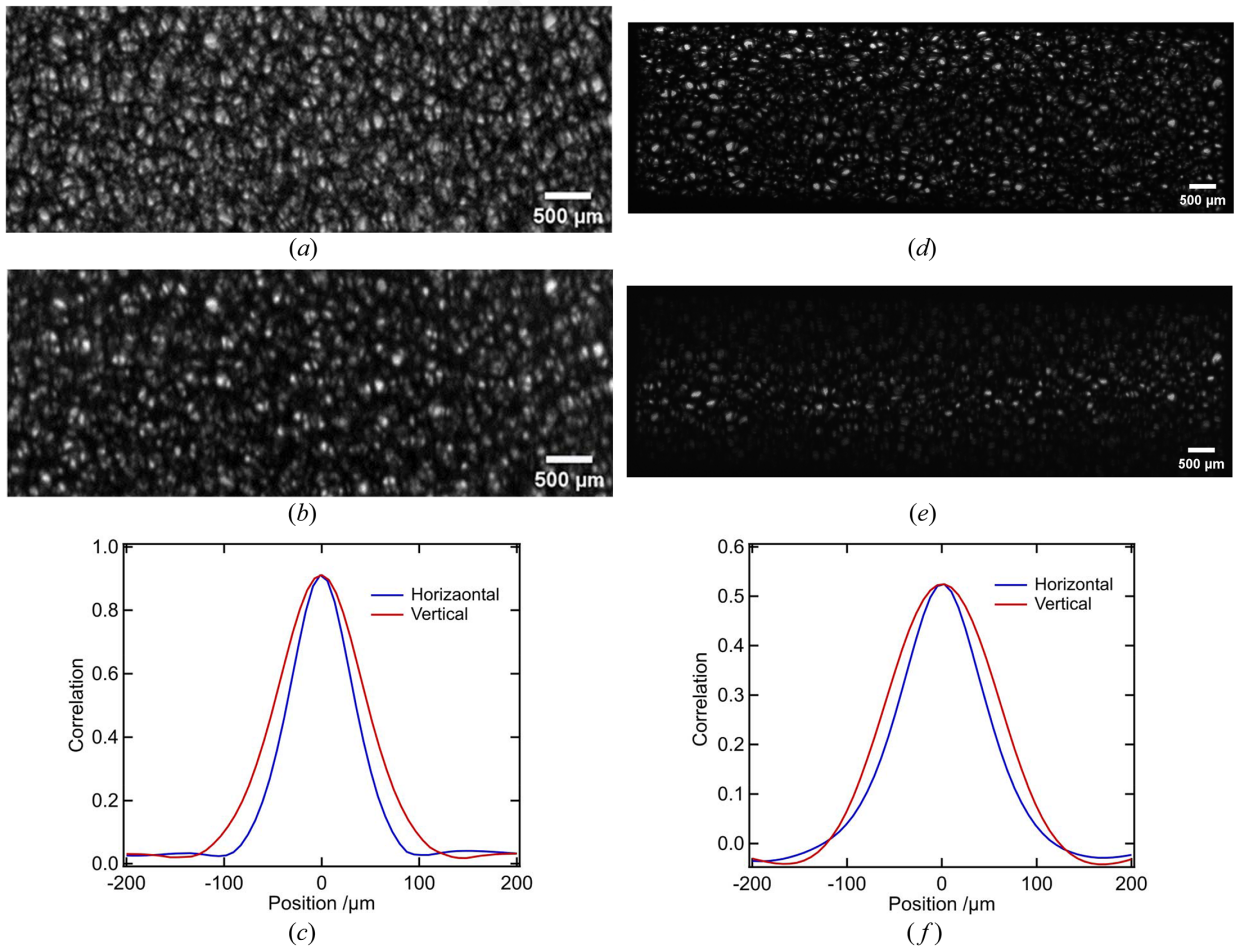
Speckle patterns with the developed beam splitter and the conventional splitter. (*a*, *b*) Transmission and diffraction speckle patterns and (*c*) correlation coefficient curves of the developed beam splitter; panels (*d*)–(*f*) correspond to the respective results of the conventional splitter.

## Appendix A Nondispersive configuration

A DuMond diagram, typically employed for crystal diffraction, is used to analyze the intrinsic connection between the energy and angular width of the incident and diffracted beams in multi-crystal configurations (Yang *et al.*, 2020 ▸; Li *et al.*, 2020 ▸). Fig. 5 ▸ shows DuMond diagrams for perfect crystal diffraction, where the dynamical diffraction effect results in the broadening of the diffracted beam angle ω_D_, which is the Darwin width of the crystal. According to the differential form of the Bragg equation,

the energy width Δλ_D_ can be determined. The blue window outlined by ω_D_ and Δλ_D_ in Fig. 5 ▸ represents the diffraction window of the crystal. It can be concluded that for white beam with parallel incidence the emitted beam will have a certain energy distribution, and for monochromatic beam with divergence the emitted beam will have a certain angular distribution. In Fig. 6 ▸, the DuMond window of the monochromator crystal remains fixed as the incident beam, while the DuMond window of the beam splitter crystal is gradually approached through angle scanning. When the two windows overlap, a diffracted intensity is detected on a detector, and the result obtained by the detector is the convolution of the two windows. In a nondispersive configuration, during the scanning process, the two DuMond windows can completely overlap, which indicates that the consistent beam-splitting fields are achieved. In comparison with the dispersive configuration in Fig. 5 ▸(*c*), following the blue arrow direction of movement, different divergences and energy of the diffracted beam are obtained, which clearly differs from the incident beam. Therefore, the nondispersive configuration shown in Fig. 6 ▸ was chosen for our experiments.

## Appendix B Developed beam splitter versus conventional splitter

By comparing with the results of the conventional splitter with a wafer shown in Figs. 6 ▸(*d*) and 6(*e*), the results from Figs. 6 ▸(*a*) and 6(*b*) suggest that the developed beam splitter exhibits high beam-splitting performance. According to Figs. 6 ▸(*d*), 6(*e*) and 6(*f*), the beam size, intensity uniformity, speckle pattern consistency and correlation coefficient achieved by the wafer splitter are all apparently inferior to that of the developed splitter [as shown in Figs. 6 ▸(*a*), 6(*b*) and 6(*c*)]. From equation (2), the correlation coefficient curves are shown in Figs. 6 ▸(*c*) and 6(*f*). The correlation coefficient of the developed beam splitter is around 0.92, while that of the wafer is only 0.52. With the same sampling rate, a higher correlation coefficient usually indicates better image quality. This means that it is difficult to achieve high-quality ghost imaging with the conventional splitter. In addition, the width of the correlation coefficient curves is reduced from approximately 104 µm (H) × 133 µm (V) to 76 µm (H) × 104 µm (V), which implies that higher spatial resolution can be achieved by the developed splitter.

## References

[bb1] Aminzadeh, L., Roberts, B., Young, B., Chiang, I. D., Svalbe, D. M., Paganin, D. M. & Kingston, A. M. (2023). *Opt. Express*, **31**, 24328–24346. 10.1364/OE.495024 37475263

[bb2] Bromberg, Y., Katz, O. & Silberberg, Y. (2009). *Phys. Rev. A*, **79**, 053840.

[bb3] Ceddia, D., Aminzadeh, A., Cook, P. K., Pelliccia, D., Kingston, A. M. & Paganin, D. M. (2023). *Optica*, **10**, 1067.

[bb4] Ceddia, D. & Paganin, D. M. (2018). *Phys. Rev. A*, **97**, 062119.

[bb5] Cheng, J. & Han, S. (2004). *Phys. Rev. Lett.* **92**, 093903. 10.1103/PhysRevLett.92.093903 15089466

[bb6] He, Y.-H., Huang, Y., Zeng, Z., Li, Y., Tan, J., Chen, L.-A., Wu, L., Li, M., Quan, B., Wang, S. & Liang, T.-J. (2021). *Sci. Bull.* **66**, 133–138. 10.1016/j.scib.2020.09.030 36654220

[bb7] He, Y.-H., Zhang, A.-X., Li, M.-F., Huang, Y.-Y., Quan, B.-G., Li, D.-Z., Wu, L.-A. & Chen, L.-M. (2020). *APL Photon.* **5**, 056102.

[bb9] Kang, Y., Yao, Y. -P., Kang, Z. -H., Ma, L. & Zhang, T. (2015). *J. Opt. Soc. Am. A*, **32**, 1063. 10.1364/JOSAA.32.001063 26367039

[bb10] Katz, O., Bromberg, Y. & Silberberg, Y. (2009). *Appl. Phys. Lett.* **95**, 131110.

[bb11] Kingston, A. M., Pelliccia, D., Rack, A., Olbinado, M. P., Cheng, Y., Myers, G. R. & Paganin, D. M. (2018). *Optica*, **5**, 1516. 10.1107/S205225251800711X PMC6038954 30002844

[bb12] Klein, Y., Sefi, O., Schwartz, H. & Shwartz, S. (2022). *Optica*, **9**, 63.

[bb13] Kunimune, Y., Yoda, Y., Izumi, K., Yabashi, M., Zhang, X.-W., Harami, T., Ando, M. & Kikuta, S. (1997). *J. Synchrotron Rad.* **4**, 199–203. 10.1107/S0909049597006912 16699230

[bb14] Kuriyama, M. & Boettinger, W. J. (1976). *Acta Cryst.* A**32**, 511–512.

[bb55] Lane, T. J. & Ratner, D. (2020). *Opt. Express*, **28**, 5898–5918. 10.1364/OE.379503 32225851

[bb15] Li, C.-B., Yin, W.-T., Jiang, H. & Zhang, Y. (2013). *Comput. Optim. Appl.* **56**, 507–530.

[bb16] Li, S., Cropp, F., Kabra, K., Lane, T., Wetzstein, G., Musumeci, P. & Ratner, D. (2018). *Phys. Rev. Lett.* **121**, 114801. 10.1103/PhysRevLett.121.114801 30265113

[bb17] Li, Z., Fan, Y.-C., Xue, L., Zhang, Z.-Y. & Wang, J. (2019). *AIP Conf. Proc.* **2054**, 060040.

[bb18] Li, Z.-L., Yang, J.-L., Si, S.-Y., Li, T., Hu, L.-F., Song, L., Xue, L., Wang, J. & Zhang, X.-W. (2020). *Nucl. Instrum. Methods Phys. Res. A*, **983**, 164526.

[bb19] Liu, H. -C. & Zhang, S. (2017). *Appl. Phys. Lett.* **111**, 031110.

[bb8] Oh, J. E., Cho, Y. W., Scarcelli, G. & Kim, Y. H. (2013). *Opt. Lett.* **38**, 682–684. 10.1364/OL.38.000682 PMC4617630 23455264

[bb20] Olbinado, M. P., Paganin, D. M., Cheng, Y. & Rack, A. (2021). *Optica*, **8**, 1538.

[bb21] Pelliccia, D., Olbinado, M. P., Rack, A., Kingston, A. M., Myers, G. R. & Paganin, D. M. (2018). *IUCrJ*, **5**, 428–438. 10.1107/S205225251800711X PMC6038954 30002844

[bb22] Pelliccia, D., Rack, A., Scheel, M., Cantelli, V. & Paganin, D. M. (2016). *Phys. Rev. Lett.* **117**, 113902. 10.1103/PhysRevLett.117.113902 27661687

[bb23] Schori, A. & Shwartz, S. (2017). *Opt. Express*, **25**, 14822–14828. 10.1364/OE.25.014822 28789065

[bb24] Shimobaba, T., Endo, Y., Nishitsuji, T., Takahashi, T., Nagahama, Y., Hasegawa, S., Sano, M., Hirayama, R., Kakue, T., Shiraki, A. & Ito, T. (2018). *Opt. Commun.* **413**, 147–151.

[bb25] Sprigg, J., Peng, T. & Shih, Y. (2016). *Sci. Rep.* **6**, 38077. 10.1038/srep38077 PMC5131481 27905498

[bb36] Wang, Z., Bovik, A. C., Sheikh, H. R. & Simoncelli, E. P. (2004). *IEEE Trans. Image Process.* **13**, 600–612. 10.1109/tip.2003.819861 15376593

[bb26] Yang, J.-L., Li, Z.-L., Li, T., Zhu, Y., Song, L., Xue, L. & Zhang, X. (2020). *Acta Phys. Sin.* **69**, 104101.

[bb27] Yu, H., Lu, R. -H., Han, S. -S., Xie, H. -L., Du, G. -H., Xiao, T. -Q. & Zhu, D. (2016). *Phys. Rev. Lett.* **117**, 113901. 10.1103/PhysRevLett.117.113901 27661686

[bb28] Zhang, A.-X., He, Y.-H., Wu, L.-A., Chen, L.-M. & Wang, B.-B. (2018). *Optica*, **5**, 374–377.

[bb29] Zhang, C., Guo, S., Cao, J., Guan, J. & Gao, F.-L. (2014). *Opt. Express*, **22**, 30063–30073. 10.1364/OE.22.030063 25606936

[bb30] Zhang, H.-P. (2023). *Investigation on Megapixel X-ray Ghost Imaging with Low Radiation Dose*, Doctorial dissertation, University of Chinese Academy of Sciences, The People’s Republic of China.

[bb31] Zhang, H.-P., Li, K., Wang, F.-X., Yu, H., Zhao, C.-Z., Du, G.-H., Li, Z.-L., Deng, B., Xie, H.-L., Han, S.-S. & Xiao, T. (2022*a*). *Chin. Opt. Lett.* **20**, 033401.

[bb32] Zhang, H. -P., Li, K., Zhao, C. -Z., Tang, J. & Xiao, T. (2022*b*). *Chin. Phys. B*, **31**, 064202.

[bb33] Zhang, H.-P., Zhao, C.-Z., Ju, X.-L., Tang, J. & Xiao, T.-Q. (2022*c*). *Acta Phys. Sin.* **71**, 074201.

[bb34] Zhao, C.-Z., Si, S.-Y., Diao, Q.-S., Hong, Z., Li, Z.-L. & Xiao, T.-Q. (2024*b*). *Proc. SPIE*, **13069**, 130691A.

[bb35] Zhao, C.-Z., Si, S.-Y., Zhang, H.-P., Xue, L., Li, Z.-L. & Xiao, T.-Q. (2022). *Acta Phys. Sin.* **71**, 046101.

[bb44] Zhao, C.-Z., Si, S.-Y., Zhang, H.-P., Xue, L., Li, Z.-L. & Xiao, T.-Q. (2024*a*). *Chin. Phys. B*, **33**, 014102.

[bb37] Zhu, H., Yu, H., Tan, Z., Lu, R., Han, S., Huang, Z. & Wang, J. (2020). *Opt. Express*, **28**, 17556–17569. 10.1364/OE.395000 32679962

